# P-1172. Characterization of Clinical Pathogens and Microbiological Outcomes for Patients Treated with IV Fosfomycin (IV-FOS) in the ZEUS Phase 2/3 Complicated Urinary Tract Infection, including Acute Pyelonephritis (cUTI/AP) Trial

**DOI:** 10.1093/ofid/ofaf695.1365

**Published:** 2026-01-11

**Authors:** Keith S Kaye, Mauricio Rodriguez, Surya Chitra, Judith N Steenbergen

**Affiliations:** Rutgers Robert Wood Johnson Medical School, New Brunswick, NJ; Meitheal Pharmaceuticals, Austin, TX; Savio Group Analytics, Hockessin, Delaware; Scientific and Medical Affairs Consulting, LLC, Washington Crossing, Pennsylvania

## Abstract

**Background:**

Approximately 3 million cUTI cases occur annually in the U.S. with attributed cost of $6 billion. Incidence of ESBL-producing Enterobacterales infections have increased. IV-FOS is a first-in-class injectable epoxide antibiotic under evaluation by the U.S. FDA for cUTI that retains *in vitro* activity against many drug-resistant strains. This post-hoc analysis was conducted to determine outcomes of patients with ESBL-producing pathogens.

**Figure:**
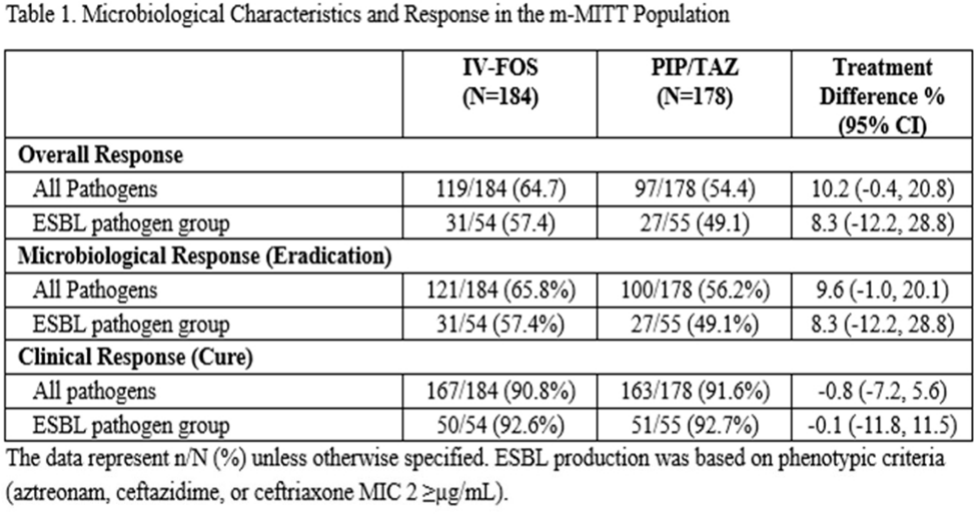

**Figure:**
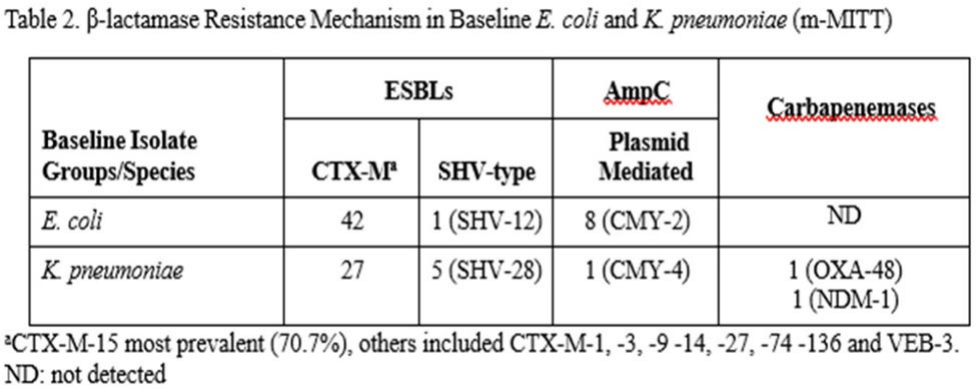

**Methods:**

Hospitalized adults with cUTI/AP were randomized 1:1 to 6 g IV-FOS q8h or 4.5 g IV piperacillin-tazobactam (PIP-TAZ) q8h for 7 days. Urine isolates that grew ≥10^3^ CFU/mL were sent to the central laboratory. ESBL production was considered positive based on aztreonam, ceftazidime, or ceftriaxone MIC ≥2 µg/mL. Outcomes in the microbiologic modified intent to treat (m-MITT) population were determined based on phenotypic criteria of ESBL production and compared to the overall cohort. The type of β-lactamase was determined for isolates that met phenotypic criteria in the IV-FOS group by whole genome sequencing.

**Results:**

Most infections were monomicrobial ( > 90%) with *E. coli* and *K. pneumoniae* accounting for > 80% of infections. Primary efficacy results demonstrated that IV-FOS was non-inferior to PIP-TAZ for the primary efficacy outcome of overall success (clinical cure and microbiologic eradication) at the test of cure (TOC) visit in the m-MITT population. Clinical response rates between arms were similar whereas microbiological eradication was demonstrated in 65.8% of IV-FOS vs. 56.2% of PIP-TAZ patients, difference 9.6 (95% CI -1.0, 20.1, Table 1). Clinical and microbiological outcomes for patients with ESBLs are in Table 1. The presence of ESBLs did not impact clinical response rates which were similar between groups. Microbiological eradication rates were lower in patients with ESBL-producing uropathogens but remained similar between groups. In the m-MITT population, 100 of 184 patients had baseline pathogens that were tested for β-lactamases. The prevalence of each β-lactamase is shown for *E. coli* and *K. pneumoniae* in Table 2.

**Conclusion:**

IV-FOS was non-inferior to PIP-TAZ in patients with cUTI/AP, including in a subset of patients with ESBLs.

**Disclosures:**

Keith S. Kaye, MD, MPH, AbbVie: Advisor/Consultant|GSK: Advisor/Consultant|Merck: Advisor/Consultant|Shionogi: Advisor/Consultant Mauricio Rodriguez, PharmD, MS-HEOR, BCCCP, BCIDP, Meitheal Pharmaceuticals: employee Judith N. Steenbergen, PhD, AcurX: Advisor/Consultant|Basilea: Advisor/Consultant|Bioversys: Advisor/Consultant|Clarametyx: Advisor/Consultant|Eagle Pharmaceuticals: Advisor/Consultant|F2G: Advisor/Consultant|Genentech: Advisor/Consultant|Innoviva: Advisor/Consultant|Meitheal: Advisor/Consultant|Melinta: Advisor/Consultant|Neuraptive: Advisor/Consultant|Neuraptive: Advisor/Consultant|Roche: Advisor/Consultant|Wockhardt: Advisor/Consultant

